# Assessing the role of vitamin D in the prevention and management of chronic rhinosinusitis: a systematic review and meta-analysis

**DOI:** 10.3389/fimmu.2026.1859469

**Published:** 2026-07-24

**Authors:** Ibrahim Sumaily, Abdulrahman A. Otaif, Khalil I. Kariri, Hussam Alshehri, Abdulelah A. Otaif, Atheer Alshammakhi, Abdullah Almutairi, Abdullah Alkhunfur, Reem Baagag, Luran Alluqmani, Abdulrahman Alelyani, Jobran M. Moshi

**Affiliations:** 1 Department of Otolaryngology, King Fahd Central Hospital, Jazan, Saudi Arabia; 2 Faculty of Medicine, Jazan University, Jazan, Saudi Arabia; 3 Department of Otolaryngology-Head & Neck Surgery, Armed Forces Hospital, Jazan, Saudi Arabia; 4 College of Medicine, King Abdulaziz University, Jeddah, Saudi Arabia; 5 General Practitioner, Qassim Health Cluster, Qassim, Saudi Arabia; 6 College of Medicine, Prince Sattam Bin Abdulaziz University, Al-Kharj, Saudi Arabia; 7 College of Medicine, Vision College, Riyadh, Saudi Arabia; 8 College of Medicine, Umm Al-Qura University, Makkah, Saudi Arabia; 9 College of Medicine, University of Tabuk, Tabuk, Saudi Arabia; 10 Department of Medical Laboratory Technology, College of Nursing and Health Science, Jazan University, Jazan, Saudi Arabia

**Keywords:** chronic rhinosinusitis, disease severity, meta-analysis, nasal polyps, quality of life, recurrence, SNOT-22, vitamin D

## Abstract

**Background:**

Chronic rhinosinusitis (CRS) is a prevalent inflammatory disorder that significantly impairs quality of life. Emerging evidence suggests that vitamin D plays an immunomodulatory role in sinonasal inflammation; however, its clinical significance in CRS remains unclear.

**Objective:**

To systematically evaluate the association between vitamin D and CRS and to assess the effects of vitamin D supplementation on disease severity, recurrence, quality of life, inflammatory markers, and postoperative outcomes.

**Methods:**

A systematic review and meta-analysis were conducted in accordance with PRISMA guidelines. A comprehensive search of PubMed, MEDLINE, Embase, and Web of Science was performed with predefined inclusion criteria registered in PROSPERO (CRD420251001899). Eligible studies included randomized controlled trials, cohort, case-control, and cross-sectional studies assessing vitamin D levels or supplementation in adults with CRS. Data were synthesized narratively, and meta-analyses were performed using a random-effects model. Methodological quality was assessed using the Newcastle-Ottawa Scale and Cochrane Risk of Bias 2 tool.

**Results:**

Twelve studies comprising 1,236 participants were included. CRS patients exhibited significantly lower vitamin D levels than controls, with deficiency rates exceeding 90% among patients with nasal polyps. Lower vitamin D levels were consistently associated with increased disease severity, including worse radiological, endoscopic, and patient-reported outcomes. Vitamin D supplementation demonstrated improvements in symptom severity, quality of life, and radiological scores, and was associated with a substantial reduction in postoperative recurrence (24.1% vs. 51.6%). Meta-analysis confirmed significantly lower serum vitamin D levels in CRS patients (MD = −12.09 ng/mL; 95% CI: −23.07 to −1.12) and a significant inverse correlation with disease severity (r = −0.52; 95% CI: −0.66 to −0.34). No statistically significant difference was observed in pooled SNOT-22 outcomes. Supplementation was well tolerated with no significant adverse effects reported.

**Conclusion:**

Vitamin D deficiency is strongly associated with increased disease severity and recurrence in CRS. Supplementation appears to provide clinical benefits and may serve as a promising adjunct to standard therapy. Further high-quality randomized controlled trials are required to establish optimal dosing strategies and inform clinical guideline recommendations.

**Systematic Review Registration:**

https://www.crd.york.ac.uk/prospero/display_record.php?RecordID=1001899, identifier CRD420251001899.

## Graphical Abstract

**Figure:**
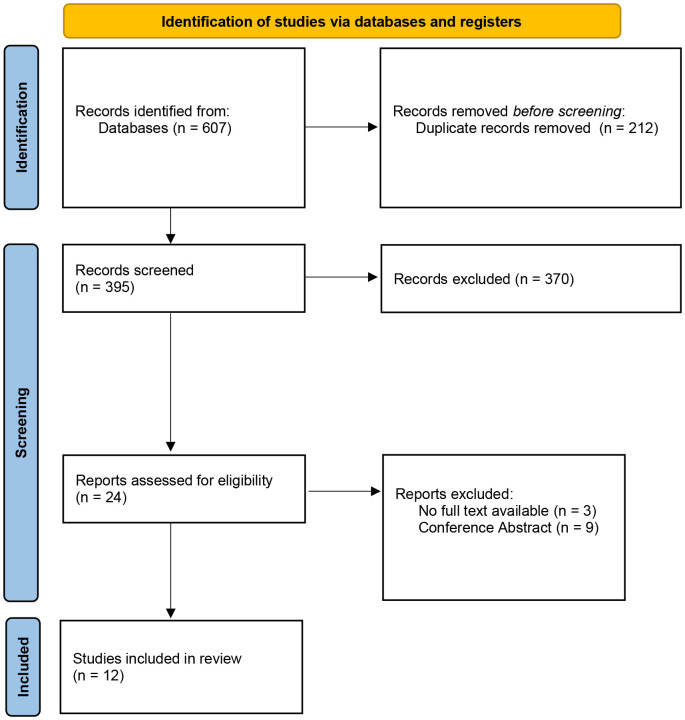
PRISMA flow diagram.

## Introduction

Chronic rhinosinusitis (CRS) is one of the most common chronic diseases worldwide. It is an upper respiratory tract ailment marked by widespread inflammation of the mucosal lining of the nose and paranasal sinuses. CRS continues to be difficult to control in many situations and it imposes a significant financial burden on society. In addition to causing a significant financial burden, CRS has a major negative impact on the lives of those affected. The effects extend beyond monetary costs; they also include limitations in day-to-day activities, mental stress, and physical discomfort. It may result in a decline in general well-being, social disengagement, and productivity. The etiology of CRS is multifaceted and includes genetic, immunological, infectious, anatomical, and biofilm-related variables. All of these pathophysiological factors inevitably result in chronic inflammation of the sinonasal mucosa (1). The prevalence estimates for CRS, according to epidemiological studies conducted worldwide, have been close to 10% when either symptoms or objective findings are considered alone. However, when the diagnostic criteria based on guidelines are followed, which require both symptoms and objective findings, the actual prevalence of CRS is consistently less than 5%. Additionally, it has been shown that nasal polyps are present in approximately one-third of those with a CRS diagnosis (2).

Two forms of CRS are distinguished: CRS with nasal polyps and CRS without polyps. Facial discomfort and loss of smell are the primary symptoms that distinguish these two types. Both symptoms have a variety of clinical manifestations and are suggestive of a substantial illness effect (3). The chronic inflammation and tissue deformation seen in CRS are believed to result from alterations in mucociliary function, defects in the sinonasal epithelial barrier, and ongoing tissue remodeling. Moreover, heightened activity of both the innate and adaptive immune systems appears to play a major role in the pathogenesis of the disease (4).

In the study of illnesses of the upper respiratory tract, the pathophysiology of vitamin D represents a noteworthy breakthrough. Numerous studies now indicate that vitamin D3 acts as an immunomodulator of both innate and adaptive immunity locally within the respiratory epithelium (5).

Multiple systematic reviews have consistently demonstrated a significant association between vitamin D deficiency and chronic rhinosinusitis (CRS), particularly in patients with nasal polyps. Al-Ebiary et al. (2018) found that patients with CRS with nasal polyps (CRSwNP) had significantly lower vitamin D levels compared to controls and those with CRS without nasal polyps (CRSsNP), while no significant difference was observed between CRSsNP patients and controls (6). Alharthi & Alzarei (2024) analyzed nine studies with 1,042 patients and reported that all studies showed a negative correlation between vitamin D levels and CRS, with a moderate inverse correlation between vitamin D levels and disease severity scores (7). Pantazidou et al. (2023) reviewed 20 studies and concluded that CRS patients, especially those with nasal polyposis, had the lowest vitamin D levels with increased disease severity (8). Stokes & Rimmer (2016) similarly found significantly lower vitamin D levels in polypoid CRS phenotypes compared to controls, often associated with increased inflammation (5).

Given the increasing evidence linking vitamin D deficiency to chronic rhinosinusitis, especially with nasal polyposis, understanding this relationship is clinically important. Vitamin D’s role as an immunomodulator suggests that low levels might contribute to ongoing inflammation, impaired mucosal healing, and worse treatment results in CRS patients. Despite extensive international research, there remains inconsistency in findings regarding the strength of this link, its effect on disease severity, and the potential benefits of supplementation. More research is necessary to clarify these relationships and support evidence-based treatment approaches. This study aims to systematically evaluate the effects of vitamin D supplementation on symptom severity, recurrence, quality of life, inflammatory markers, and postoperative outcomes in adults with chronic rhinosinusitis.

## Methods

### Databases and search strategy

Comprehensive searches were conducted across four major electronic databases: PubMed, MEDLINE (via Ovid), Embase, and Web of Science. The searches were conducted without date restrictions and limited to English-language publications. Search terms combined controlled vocabulary, such as MeSH and Emtree terms, with free-text keywords related to “chronic rhinosinusitis”, “nasal polyps”, and “vitamin D” or “25-hydroxyvitamin D”. Boolean operators (AND/OR) were used to improve sensitivity and specificity. The search strategy was tailored to each database. Additionally, the reference lists of included studies and relevant reviews were manually checked for further eligible publications. Only published, peer-reviewed articles were considered, with grey literature, conference abstracts, case reports, and letters excluded.

### Inclusion and exclusion criteria

Eligibility criteria were prespecified in the PROSPERO registration (CRD420251001899). Studies were included if they involved adults (≥18 years) diagnosed with chronic rhinosinusitis (CRS), with or without nasal polyps, and evaluated vitamin D either as supplementation (oral or intranasal) or through measurement of serum 25-hydroxyvitamin D levels. Eligible comparators included placebo, standard CRS treatment without vitamin D, or healthy control groups. Studies were required to report at least one clinically relevant outcome, including symptom severity, recurrence rates, quality of life, inflammatory markers, serum vitamin D levels, or postoperative outcomes. Accepted study designs comprised randomized controlled trials, cohort studies, case-control studies, and cross-sectional studies. Studies were excluded if they involved pediatric populations or patients with systemic or autoimmune conditions unrelated to CRS, lacked a comparator group, or were non-original research such as case reports, reviews, conference abstracts, editorials, or letters. Non-human or *in vitro* studies, and studies addressing acute sinusitis or fungal sinusitis that did not meet CRS diagnostic criteria, were also excluded.

### Outcome measurement

The primary outcome was symptom severity, assessed using validated instruments such as the Sino-Nasal Outcome Test-22 (SNOT-22), Total Nasal Symptom Score (TNSS), or Visual Analogue Scale (VAS). Secondary outcomes included recurrence rates of CRS or the need for revision surgery, quality of life assessed using validated tools such as SNOT-22 or SF-36, inflammatory biomarkers including IL-4, IL-5, IL-6, TNF-α, CRP, IgE, RANTES, and bFGF, as well as radiological and endoscopic outcomes such as Lund-Mackay CT scores and Lund-Kennedy endoscopic scores. Additional outcomes included serum vitamin D levels before and after supplementation, and any reported adverse events associated with vitamin D use.

### Data synthesis and quality assessment

Two reviewers independently extracted data using a predefined template that included study characteristics, participant demographics, methods, vitamin D assessment levels, outcome measures, and details of any intervention.

Given the variability in study designs, interventions, outcome measures, and methods for assessing vitamin D, a combined qualitative and quantitative synthesis approach was adopted. A narrative synthesis was performed to summarize findings across all included studies.

Where sufficient homogeneous data were available, a meta-analysis was conducted in accordance with PRISMA guidelines to evaluate the association between vitamin D and chronic rhinosinusitis (CRS). Studies reporting continuous outcomes as mean ± standard deviation or correlation coefficients were included. Pooled effect sizes were calculated as mean difference (MD) or standardized mean difference (SMD) with 95% confidence intervals using a random-effects model. Correlation coefficients were transformed using Fisher’s Z transformation before pooling. Statistical heterogeneity was assessed using the I² statistic and Cochran’s Q test, and sensitivity analyses were performed to explore potential sources of heterogeneity. Publication bias was evaluated using funnel plot. All statistical analyses were performed using R software (R Foundation for Statistical Computing, Vienna, Austria) with the ‘metafor’ and ‘meta’ packages.

The methodological quality of included studies was independently assessed by two reviewers using design-specific tools. Observational studies were evaluated using the Newcastle-Ottawa Scale (NOS), which assesses the selection, comparability, and outcome domains. Studies scoring 7–9 stars were considered high quality, 4–6 stars moderate quality, and <4 stars low quality. Randomized controlled trials were assessed using the Cochrane Risk of Bias 2 (RoB 2) tool, which evaluates bias across the domains of randomization, deviations from intended interventions, missing outcome data, outcome measurement, and selective reporting. Each domain was classified as low risk, some concerns, or high risk. Discrepancies between reviewers were resolved through discussion or, when necessary, consultation with a third reviewer.

Clinical heterogeneity was assessed based on differences in study populations, vitamin D supplementation regimens, outcome assessment tools, and duration of follow-up.

## Results

### Study search and selection

**Figure 1:**
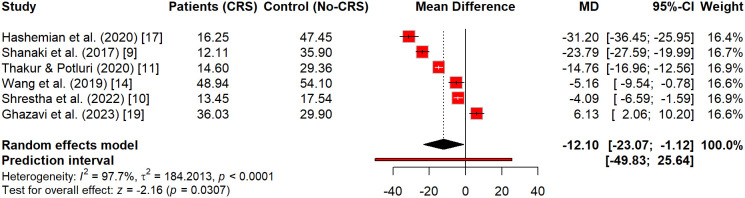
Comparison of vitamin D Level (ng/mL) between CRS patients and comparison group.

Our search yielded 607 records, from which 212 duplicates were removed using reference management software, resulting in 395 distinct articles for review analysis. During the initial screening of titles and abstracts, 370 articles were excluded because they did not meet the inclusion criteria of this review. Twenty-five citations advanced to full-text eligibility; one was a duplicate of a previously included study, narrowing the group to 24 manuscripts for review. Twelve records were excluded: three lacked complete texts despite author outreach and interlibrary loan requests, and nine were conference abstracts that offered insufficient detail for a quantitative synthesis. Ultimately, 12 studies that met the inclusion criteria were included in this review (**Figure 1**).

## Quality assessment of included studies

### Appraisal of observational research

The methodological rigor of the nine observational studies was assessed using the Newcastle-Ottawa Scale (NOS). This instrument addresses three domains: selection (max score: 5 stars), comparability (max score: 2 stars), and outcome assessment (max score: 3 stars), for a maximum aggregated score of 10 points. An overall score of 7 or more was deemed high quality, 4–6 was considered moderate, and < 4 was classified as low quality. Of the nine studies, eight (88.9%) had high methodological quality, accumulating 7–9 points. Three investigations attained the top score of 9, reflecting an effectively balanced performance across domains (9–11). They applied clearly defined selection criteria, received maximal points, attained full comparability ratings, and incorporated a thorough outcome assessment, justifying the three-star maximum.

Five studies were high performers, scoring between 7 and 8 points and maintaining high-quality ratings (12–14). each secured an 8 out of 10, while (15) received a 7 (16). also rated 8, gaining only a 1-star rating for comparability; this shortfall was offset by excellent ratings in selection and outcome. The only moderate score (6 out of 10) was for (1) because of a 1-star rating for comparability and a 2-star rating for outcome assessment. The excellent quality observed in the observational studies was especially pronounced in the selection domain: seven studies earned four stars, and only two earned three stars. All studies ensured adequate comparability by controlling for relevant confounders, although the depth of the adjustment varied. The assessment of outcomes was generally rigorous, with six studies attaining perfect scores (**Table 1**).

**Table 1:** Quality assessment of included observational studies using newcastle-ottawa scale.

Study	Selection (★/5)	Comparability (★/2)	Outcome (★/3)	Total score	Quality rating
Thakur & Potluri, 2020 (11)	★★★★	★★	★★★	9/10	High
Bavi et al., 2019 (12)	★★★★	★★	★★	8/10	High
Shanaki et al., 2017 (9)	★★★★	★★	★★★	9/10	High
Shrestha et al., 2022 (10)	★★★★	★★	★★★	9/10	High
Zand et al., 2020 (15)	★★★	★★	★★	7/10	High
Kalińczak-Górna et al., 2021 (13)	★★★	★★	★★★	8/10	High
Sansoni et al., 2015 (16)	★★★★	★	★★★	8/10	High
Baruah et al., 2020 (1)	★★★	★	★★	6/10	Moderate
Wang et al., 2018 (14)	★★★★	★★	★★	8/10	High

### Assessment of randomized controlled trials

Three randomized controlled trials were assessed using the Cochrane Risk of Bias 2 (RoB 2) tool, which evaluates five domains: randomization process, deviations from intended interventions, missing outcome data, measurement of the outcome, and selection of the reported result. Hashemian et al. (2020) demonstrated the highest methodological rigor, achieving low risk of bias across all five domains. This study employed appropriate randomization procedures, maintained protocol adherence, reported complete outcome data, utilized validated measurement tools, and comprehensively presented pre-specified outcomes (17). Two RCTs were rated as having “some concerns” regarding the overall risk of bias. Kammili et al. (2024) showed a low risk in four domains but raised some concerns regarding deviations from the intended interventions, suggesting potential issues with protocol adherence or per-protocol analysis (18). Ghazavi et al. (2023) demonstrated a low risk in four domains but exhibited some concerns related to missing outcome data, indicating potential attrition bias or incomplete follow-up that could influence the interpretation of results (19) (**Table 2**).

**Table 2:** Quality assessment of RCTs using cochrane risk of bias 2 (RoB 2) tool.

Study	Domain 1: randomization	Domain 2: deviations	Domain 3: missing data domain	4: Outcome measurement	Domain 5: selective reporting	Overall risk
Kammili et al. (2024) (18)	Low risk	Some concerns	Low risk	Low risk	Low risk	Some concerns
Hashemian et al. (2020) (17)	Low risk	Low risk	Low risk	Low risk	Low risk	Low risk
Ghazavi et al. (2023) (19)	Low risk	Low risk	Some concerns	Low risk	Low risk	Some concerns

### Demographic characteristics of included studies

This systematic review synthesized 12 investigations involving 1,236 participants diagnosed with chronic rhinosinusitis (CRS) or classified as healthy controls. Data were drawn from studies conducted on several continents, although the predominant setting was Asia, which contributed ten of the included investigations: four from India (1, 10, 11, 18), five from Iran (9, 12, 15, 17, 19), and one from China (14). One study was reported from Poland (13) and one from the United States (1) (**Table 3****).**

**Table 3:** Demographic characteristics of included studies.

Author ID, country, year	Overall study demographics			Age	Gender distribution	CRS phenotype distribution	Comorbidities and allergic status	Geographic and environmental characteristics
	Total sample size	Study groups	Study design					
Thakur & Potluri, India, 2020 (11)	91	60 CRS cases (30 CRSwNP + 30 CRSsNP) and 31 controls	Prospective case-control study	Mean Age (years): • CRS overall: 32.78 ± 7.57 (range 19-48) • CRSwNP: 31.50 ± 7.68 (range 19-45) • CRSsNP: 34.07 ± 7.35 (range 22-48) • Controls: 30.32 ± 4.14 (range 25-43) Age inclusion: >18 years	CRS cases: • 26 females (43.33%) • 34 males (56.66%) Controls: • 14 females (45.16%) • 17 males (54.84%)	• 30 CRSwNP (50%) • 30 CRSsNP (50%)	Allergic Status • 26 cases (43.33%) with positive allergy history • 34 cases (56.67%) non-allergic Exclusions: • Bronchial asthma • Smoking • Pregnancy • Systemic conditions affecting vitamin D • Malabsorption syndromes • Renal, GI, endocrine, skeletal disorders	Altitude: • Study population residing >2000m above sea level • Center serves population at 1500-3300m Location: • Himachal Pradesh, India (high altitude region) Environmental factors: • Vitamin D measurements during maximum sunshine months (April-June) • Region has >200 hours of monthly sunshine throughout the year
Kammili et al., India, 2024 (18)	50	• 25 vitamin D3 group • 25 placebo group	Prospective randomized controlled trial	Age inclusion: 18–60 years Mean Age (years): • Vitamin D3 group: 34.7 ± 5.2 • Placebo group: 36.5 ± 6.94	Total: • 28 males (56%) • 22 females (44%) Vitamin D3 group: • 13 males • 12 females Placebo group: • 15 males • 10 females	• 50 CRSsNP (100%) • 0 CRSwNP Note: Patients with nasal polyps were excluded	Allergic Rhinitis: • 19 patients (38%) with AR • 31 patients (62%) without AR Exclusions: • Nasal polyps • Previous sinus surgery • Smoking • Alcohol abuse • Pregnancy • Prolonged steroid use • Systemic disorders (endocrine, renal, skeletal) • Neoplasms • Vitamin D3 sensitivity	Location: • Primary healthcare center in India Study Period: • December 2021 to December 2023 Other: • All participants had vitamin D deficiency (<20 p<p<) at baseline • Standard treatment included fluticasone nasal spray, antibiotics, and montelukast
Bavi et al., Iran, 2019 (12)	338	Study Groups: • 166 CRSwNP cases • 172 healthy controls	Cross-sectional study	Mean Age (years): • CRSwNP: 41.04 ± 13.0 (95% CI: 39.05-43.05) • Controls: 41.01 ± 14.8 (95% CI: 38.77-43.24) Age inclusion: Not specifically stated	CRSwNP: • 98 males (59%) • 68 females (41%) Controls: • 93 males (54%) • 79 females (46%)	• 166 CRSwNP (100%) • 0 CRSsNP Note: All cases had eosinophilic nasal polyposis	Allergy: • 55 patients (33%) with allergy • 111 patients (67%) without Asthma: • 52 patients (31%) with asthma Smoking: • 82 CRSwNP patients (49.4%) • 17 controls (9.9%) Exclusions: • Vitamin D supplementation • Anticonvulsant use • OCP usage • Corticosteroid use (last 3 months) • Chronic diseases • Malnutrition	Location: A large city in northeastern Iran • Almost always sunny (but polluted) • Tertiary hospitals (Qaem and Imam Reza) Study Period: • October 2015 to August 2017
Shanaki et al., Iran, 2017 (9)	89	Study Groups: • 45 CRSwNP patients • 44 healthy controls (45 recruited, 1 with missing lab data)	Age-gender matched case-control study	Age inclusion: 18–65 years Mean Age (years): • Overall: 43.2 ± 6.9 • CRSwNP: 41.04 ± 13.0 • Controls: 41.00 ± 14.8	Both groups: • 23 females (51%) • 22 males (49%) Gender matched between groups	• 45 CRSwNP (100%) • 0 CRSsNP	Exclusions: • CRS without polyps • Liver or kidney disease • Infectious diseases • Malignancies • Allergic and immunological diseases • Immunosuppressive medications • Vitamin D or antioxidant supplements • Smoking Note: All participants were free from allergic conditions	Location: • Tehran, Iran Setting: • Loghman-Hakim Hospital • Shahid Beheshti University of Medical Sciences Study Period: • June 2015 to June 2016
Shrestha et al., India, 2022 (10)	90	Study Groups: • 30 Primary Nasal Polyposis (PNP) - not operated prior • 30 Recurrent Nasal Polyposis (RNP) - operated prior • 30 healthy controls	Prospective case-control study	Age inclusion: >14 years Mean Age (overall): 43.2 ± 6.9 years Note: Specific age data for individual groups was not provided	Not specified	• 30 PNP (33.3%) • 30 RNP (33.3%) All CRSwNP (chronic rhinosinusitis with nasal polyposis)	Exclusions: • Osteomalacia • Thyroid dysfunction • Rheumatoid arthritis • Other illnesses affecting vitamin D & interleukin levels Note: Allergic status not specified	Location: • AIIMS, New Delhi, India • Department of Otorhinolaryngology< Context: • Indian population with high prevalence (70-100%) of vitamin D deficiency in healthy individuals
Hashemian et al., Iran, 2020 (17)	40	Study Groups: • 20 vitamin D3 group (4000 IU daily) • 20 placebo group Note: Initially 46 patients were randomized; 6 were excluded during follow-up	Triple-blind placebo-controlled randomized clinical trial	Age inclusion: >18 years Mean Age (years): • Vitamin D3: 41.35 ± 13.58 • Placebo: 42.05 ± 13.79 Overall mean: ~41.7 years	Vitamin D3 group: • 16 males (80%) • 4 females (20%) Placebo group: • 12 males (60%) • 8 females (40%)	• 40 CRSwNP (100%) • 0 CRSsNP All patients had bilateral nasal polyposis	AERD: • 3 patients (15%) in each group Revision Surgery: • 2 patients (10%) in each group Inclusion: • Vitamin D3 insufficiency (10–30 ng/mL) • Failed standard medical treatment Exclusions: • Smokers • Pregnancy • Long-term systemic steroids • Systemic disorders • Various medications	Location: • Besat Hospital, Hamadan, Iran Study Period: • September 2016 to November 2017 Context: • All patients underwent FESS (functional endoscopic sinus surgery) • Post-surgery routine treatment included fluticasone spray, irrigation, antibiotics, and montelukast
Zand et al., Iran, 2020 (15) Iran	93	• 93 CRS w NP cases	Prospective cross-sectional study	Age inclusion: 18–65 years Mean Age (years): • Overall: 37.7 ± 13.6 (range 18-65) • Males: 23.3 ± 11.5 • Females: 23.5 ± 19.1	Total: • 55 males (59.1%) • 38 females (40.9%)	• 93 CRSwNP (100%) • 0 CRSsNP Note: All cases had chronic rhinosinusitis with nasal polyposis	Smoking status: • Data collected but no significant difference between smokers and non-smokers (P = 0.272) Allergy status: • Data collected but no significant difference in vitamin D levels based on allergy status (P = 0.340) Exclusions: • Systemic steroids use (1 year prior) • Vitamin D supplements (1 year prior) • Chronic kidney disease • Chronic liver disease • Granulomatous disease • Sarcoidosis	Location: • Yazd, Iran • ENT clinics of Yazd Study Period: • 2016-2017 Setting: • Ear, nose, and throat clinics • Shahid Sadoughi University of Medical Sciences Environmental factors: • Mean serum vitamin D level: 24.6 ± 16.9 ng/mL<br>• Study assessed vitamin D deficiency in relation to disease severity
Kalińczak-Górna et al., Poland, 2021 (13)	40	• Group 1: 20 patients (≤10 points on Lund-Mackay scale) • Group 2: 20 patients (>10 points on Lund-Mackay scale)	Prospective observational study	Age inclusion: Not specified Mean Age: Not provided in demographic tables	Not specified	• Both CRSwNP and CRSsNP included From **Table 5**: • CRSwNP: mean vitamin D 20.36 ng/mL pre-op • CRSsNP: mean vitamin D 27.68 ng/mL pre-op Note: Exact distribution not specified	Exclusions: • Other systemic diseases (metabolic, cardiovascular) • Neoplasms • Previous surgery (within 6 months) • Vitamin D supplementation Note: Atopy/asthma status not specified	Location: • Bydgoszcz, Poland Setting: • Department of Otolaryngology, Collegium Medicum, Nicolaus Copernicus University • University Hospital Study Period: • Not specified Climate: • Temperate climate mentioned
Sansoni et al., USA, 2015 (16)	57	• 31 CRSsNP cases • 14 CRSwNP cases • 12 controls	Prospective case-control study	Age inclusion: Adults undergoing ESS Mean Age (years): • Overall: 48.7 ± 18.9 • No specific age data provided by group	Total: • 26 males (45.6%) • 31 females (54.5%) Note: Gender distribution by CRS subtype not specified	• 31 CRSsNP (54.4%) • 14 CRSwNP (24.6%) Note: All CRSwNP had nasal polyposis	Exclusions: • Oral steroids (within 4 weeks) • Immunotherapy (within 4 weeks) • Rheumatoid arthritis • Immunodeficiency • Cystic fibrosis • AFRS and Samter’s triad included in CRSwNP group Note: Allergic status not specified	Location: • Oregon Health & Science University, Portland, and • Medical University of South Carolina, Charleston, SC Setting: • Two tertiary academic medical centers in USA Study Period: • January 2012 to August 2014
Ghazavi et al., Iran, 2023 (19)	60	• 29 vitamin D3 group • 31 placebo group Note: Initially 70 patients (35 per group); 10 excluded due to lack of follow-up	Randomized controlled trial	Age inclusion: 18–64 years Mean Age (years): • Vitamin D3 group: 45.07 ± 14.79 • Placebo group: 40.61 ± 9.71	Vitamin D3 group: • 17 males (58.6%) • 12 females (41.4%) Placebo group: • 15 males (48.4%) • 16 females (51.6%)	• 60 CRSwNP (100%) • 0 CRSsNP Note: All patients underwent endoscopic sinus surgery for CRSwNP	Inclusion: • Vitamin D deficiency (<20 ng/mL) or insufficiency (21–29 ng/mL) Exclusions: • Samter’s syndrome • Nasal masses • Previous vitamin D treatment • Smoking • Rickets, osteoporosis, osteomalacia • Other metabolic bone diseases Note: Allergic status not specified	Location: • Isfahan, Iran Setting: • Kashani and Alzahra hospitals • Isfahan University of Medical Sciences Study Period: • April 2019 to December 2019 Other: • All patients received postoperative fluticasone spray and saline irrigation
Baruah et al., India, 2020 (1)	200	• 100 vitamin D group (Cholecalciferol 60000IU weekly for 3 months) • 100 placebo group	Retrospective 1-year study	Age inclusion: 18–60 years (inferred) Mean Age (years): • Vitamin D group: 33.21 (range 15-59) • Placebo group: 34.26 (range 15-60)	Vitamin D group: • 66 females (66%) • 34 males (34%) Placebo group: • 58 females (58%) • 42 males (42%)	• Study focused on CRS patients Note: No specific breakdown between CRSwNP and CRSsNP provided	Inclusion: • CRS patients with vitamin D deficiency (<20 ng/mL) Exclusions: • Pregnancy and lactation • Patients taking multivitamins with vitamin D (≥6 months) • Systemic steroids or NSAIDs (≥3 months) • Nephrolithiasis • Chronic renal disease • Severe liver disease Note: Allergic status not specified	Location: • Tata Main Hospital, Jamshedpur, Jharkhand, India Setting: • ENT OPD (Outpatient Department) Study Period: • January 2019 to December 2019 Other: • All patients had vitamin D deficiency at baseline (mean 12.31 ng/mL in treatment group)
Wang et al., China, 2019 (14)	88	• 42 CRSwNP • 25 CRSsNP • 21 controls (nasal septum deviation without inflammatory disease)	Retrospective analysis of prospectively collected data	Age inclusion: 18–72 years Mean Age (years): • Overall: 43 • CRSwNP: 46.50 ± 14.68 • CRSsNP: 41.56 ± 15.54 • Control: 39.61 ± 12.67	Total: • 46 males (52.3%) • 42 females (47.7%) CRSwNP: • 21 males (50%) • 21 females (50%) CRSsNP: • 12 males (48%) • 13 females (52%) Control: • 13 males (61.9%) • 8 females (38.1%)	• 42 CRSwNP (47.7%) • 25 CRSsNP (28.4%) • 21 controls (23.9%)	Asthma: • CRSwNP: 5/42 (11.9%) • CRSsNP: 3/25 (12%) • Control: 2/21 (9.5%) Atopy: • CRSwNP: 9/42 (21.4%) • CRSsNP: 7/25 (28%) • Control: 5/21 (23.8%) Smoking: • CRSwNP: 8/42 (19%) • CRSsNP: 6/25 (24%) • Control: 3/21 (14.3%) Exclusions: • Daily vitamin D supplements • Oral steroids/immunomodulatory agents (within 30 days) • Disorders affecting vitamin D metabolism • Pregnancy	Location: • Hangzhou, China Setting: • First Affiliated Hospital, School of Medicine, Zhejiang University Study Period: • Not specified Other: • Vitamin D deficiency prevalent: 93% in CRSwNP, 56% in CRSsNP, 38% in controls • Mean BMI: CRSwNP 25.43 ± 6.14, CRSsNP 23.77 ± 2.72, Control 23.13 ± 2.84

### Study design and sample sizes

This synthesis encompassed a collection of studies utilizing different designs: five adopted a prospective case-control format (9–11, 13, 16), three utilized a randomized controlled trial framework (17, 19), two were cross-sectional (12, 15), and two relied on a retrospective cohort design (1, 14). The cohorts formed within these studies exhibited considerable variability, with a minimum enrollment of 40 (13, 17) and a maximum of (12) (**Table 3****).**

### Age and gender characteristics

The eligibility age limits varied between studies, although most stipulated a minimum age of 18 years. Within the pooled cohort, the mean age ranged from 30 to 48 years. Thakur and Potluri (2020) documented a mean age of 32.78 ± 7.57 years in a cohort with CRS (11), whereas Sansoni et al. (2015) reported an overall mean age of 48.7 ± 18.9 years (16). The gender ratio was mostly homogeneous; males accounted for 45.6% to 61.9% across most reviews. The largest cohort, published by Bavi et al. (2019), reported 59% of CRSwNP cases and 54% of control subjects were men (12) (**Table 3****).**

### CRS phenotype distribution

The distribution of CRS phenotypes across the reviewed literature showed significant heterogeneity. Seven studies specifically analyzed cohorts of chronic rhinosinusitis with nasal polyps (CRSwNP) (1, 9, 10, 12, 15, 17, 19), whereas one study examined individuals with chronic rhinosinusitis without nasal polyps (CRSsNP) (18). The remaining four reports presented mixed cohorts: Thakur and Potluri (2020) allocated equal numbers of CRSwNP and CRSsNP patients (n = 30 per group) (11); Sansoni et al. (2015) recorded 31 CRSsNP and 14 CRSwNP subjects (16); Wang et al. (2019) examined 42 CRSwNP and 25 CRSsNP (14); and Kalińczak-Górna et al. (2021) recruited subjects of both phenotypes without disclosing specific numeric ratios (13) (**Table 3****).**

### Comorbidities and allergic status

Reports of allergic rhinitis among patients with CRS show considerable variation, ranging between 33% (12) and 43.33% (11). The co-occurrence of asthma among patients with chronic rhinosinusitis with nasal polyps (CRSwNP) ranges from 11.9% to 31.0% in published studies (12, 14). Smoking behavior was observed in several analyses, and most randomized controlled trials enforced a no-smoking enrolment rule (17, 19). In complementary observational research, smoking rates were recorded as 9.9% in the control group and up to 49.4% in the CRSwNP series (12) (**Table 3****).**

### Geographic and environmental characteristics

Relevant environmental exposures include the presence of high-altitude communities in India living at elevations above 2000 m (11) and the assessment of vitamin D levels acquired during peak sunlight months. Iranian-based studies sampled multiple urban centers, including Tehran (9), Hamadan (17) (15),, and Isfahan (19) to reflect a spectrum of local climates. Low vitamin D status, defined as levels below 20 ng/mL, has consistently served as a selection benchmark in intervention studies (1, 18, 19). **Table 3** presents a summary of the demographic characteristics of the included studies (**Table 3****).**

### Primary outcomes - symptom severity

This systematic review identified 12 studies that examined the correlation between vitamin D levels and symptoms of CRS. These studies demonstrated consistent and significant inverse relationships between serum 25-hydroxyvitamin D levels and disease severity markers. Moreover, intervention studies have demonstrated significant clinical improvement following vitamin D supplementation.

### Vitamin D deficiency and disease severity associations

#### Radiological and endoscopic severity

Several observational studies have found a strong negative correlation between vitamin D levels and various measures of disease severity and complexity. Thakur and Potluri (2020) reported significant inverse correlations between serum vitamin D and Lund-Mackay CT scores (r = -0.604, p < 0.001) and Lund-Kennedy endoscopic scores (r = -0.595, p < 0.001), with CRS patients having significantly lower vitamin D levels (14.60 ± 7.68 ng/mL) than the control group (29.36 ± 7.49 ng/mL) (11). These findings were corroborated by Bavi et al. (2019), who reported even stronger correlations with CT scores (r = -0.66, p < 0.0001) and endoscopic scores (r = -0.71, p < 0.0001) and reported vitamin D deficiency as an independent predictor of CRS (OR = 0.69) (12) (**Table 4****).**

**Table 4:** Primary outcome – symptom severity.

Author ID, country, year	Vitamin D sssessment and interventions	Symptoms and severity measures	Key findings on vitamin D effect
Thakur & Potluri, India, 2020 (11)	Serum 25-OH vitamin D levels (Case-control comparison	• Lund-Mackay CT scores • Lund-Kennedy endoscopic scores	Association with Severity: • Vitamin D inversely correlated with CT scores (r=-0.604, p<0.001) • Vitamin D inversely correlated with endoscopic scores (r=-0.595, p<0.001) • Lower vitamin D = worse disease severity • CRS patients: 14.60 ± 7.68 ng/mL vs Controls: 29.36 ± 7.49 ng/mL
Kammili et al., India, 2024 (18)	Intervention: 400 IU/day for 1 month vs placebo	• SNOT-22 scores • Lund-Mackay CT scores	Treatment Effect: • SNOT-22 improved 34.4% with supplementation vs 22.8% placebo (p<0.0001) • CT scores improved 53.7% with supplementation vs 27.6% placebo (p<0.0001) • Significant symptom and radiological improvement with vitamin D supplementation
Bavi et al., Iran, 2019 (12)	Serum 25-OH vitamin D levels (Case-control comparison)	• SNOT-22 scores • Lund-Mackay CT scores • Lund-Kennedy scores	Association with Severity: • Strong negative correlation with CT scores (r=-0.66, p<0.0001)<br>• Strong negative correlation with endoscopic scores (r=-0.71, p<0.0001) • Moderate negative correlation with SNOT-22 (r=-0.49, p<0.001) • Vitamin D deficiency is an independent predictor of CRS (OR = 0.69)
Shanaki et al., Iran, 2017 (9)	Serum 25-OH vitamin D levels (Case-control comparison)	Clinical severity assessment	Disease Association: • CRSwNP patients had 36.27% lower vitamin D than controls (p<0.001) • Mean levels: CRSwNP 12.11 ± 6.27 vs Controls 35.90 ± 17.19 ng/mL • No correlation with vitamin D binding protein levels
Shrestha et al., India, 2022 (10)	Serum 25-OH vitamin D levels (Case-control comparison)	• SNOT-22 scores • Lund-Mackay CT scores • Inflammatory markers (IL-4, IL-5, IL-13)	Disease Association: • Vitamin D deficiency in 90% of primary and 100% of recurrent polyposis • Mean vitamin D: Primary 13.45 ± 9.12, Recurrent 12.4 ± 8.47, Controls 17.54 ± 7.94 ng/mL (p<0.05) • Lower vitamin D associated with higher inflammatory markers
Hashemian et al., Iran, 2020 (17)	Intervention: 4000 IU/day for 1 month vs placebo	• SNOT-22 scores • Meltzer endoscopic grading scores	Treatment Effect: • 6 months post-intervention, severity was significantly lower in the VD3 group • SNOT-22: 16.25 ± 10.16 (VD3) vs. 47.45 ± 13.55 (placebo); P<0.001 • Meltzer scores: 0.50 ± 0.60 (VD3) vs. 2.65 ± 0.93 (placebo); P<0.001 • No adverse effects observed
Zand et al., Iran, 2020 (15) Iran	Serum 25-hydroxyvitamin D levels (Case-control comparison)	• SNOT-22 scores • Lund-Mackay CT scores	Association with Severity • Negative correlation between SNOT-22 and serum vitamin D (P = 0.034) • Negative correlation between Lund-Mackay scores and serum vitamin D (P = 0.027) • Direct relationship between LMS and SNOT-22 (P<0.0001) • Mean vitamin D: 24.6 ± 16.9 ng/mL
Kalińczak-Górna et al., Poland, 2021 (13)	Serum vitamin D levels before and after FESS procedure	• SNOT-20 questionnaire • Lund-Mackay scale • Lund-Kennedy scale	Association with Severity: • Lower VD3 levels in patients with higher disease stage • Group 2 (severe): 23.01 ng/mL vs Group 1 (mild): 28.02 ng/mL before surgery (p<0.05) • Post-surgery VD3 increased: Group 1 to 37.98 ng/mL, Group 2 to 27.67 ng/mL • Reduction of inflammation increases VD3 levels
Sansoni et al., USA, 2015 (16)	Plasma 25-VD3 levels (measured by ELISA)	• MCP-1 levels • RANTES levels • bFGF levels in sinonasal mucus	Association with Inflammatory Markers: • Significant negative correlation with RANTES in CRSwNP (r = −0.612; p = 0.026) • Significant negative correlation with bFGF in CRSwNP (r = −0.578; p = 0.039) • No significant correlation in CRSsNP patients • VD3 may regulate RANTES and bFGF expression in CRSwNP
Ghazavi et al., Iran, 2023 (19)	Intervention: 50,000 IU once weekly for 8 weeks vs placebo	• SNOT-22 scores • Nasal polyp recurrence rate	Treatment Effect: • SNOT-22 reduction significantly higher with VD supplementation (36.03 ± 10.71 vs. 29.90 ± 11.99; P = 0.041) • Lower polyp recurrence with VD: 24.1% vs 51.6% in controls • OR for recurrence: 0.298 [0.099–0.900]; P = 0.032 • VD supplementation reduces symptom severity and recurrence
Baruah et al., India, 2020 (1)	Serum 25-OH vitamin D levels (ELISA) Intervention: Cholecalciferol 60000IU weekly × 3 months vs placebo	• Total Nasal Symptom Score (TNSS)	Treatment Effect: • Baseline vitamin D: 12.31 ng/mL → post-treatment: 29.71 ng/mL • TNSS improved from 11.94 to 0.33 with vitamin D vs 11.96 to 2.29 with placebo (p<0.0001) • 93% of CRS patients had vitamin D deficiency (<20 ng/mL) • Significant symptom improvement with supplementation
Wang et al., China, 2019 (14)	Serum 25-hydroxyvitamin D3 levels (ELISA) Case-control comparison	• Lund-Mackay CT scores • SNOT-22 scores • Postoperative improvement (6 months)	Association with Severity: • CRSwNP: 38.2 ± 9.1 nmol/L vs CRSsNP: 48.94 ± 12.1 nmol/L vs Controls: 54.1 ± 17.1 nmol/L (p<0.001) • Vitamin D inversely correlated with SNOT-22 (r=-0.378, p=0.013) • No correlation with Lund-Mackay score (r=-0.240, p=0.126) • Vitamin D positively correlated with postoperative improvement (r=0.567, p<0.001) • 93% of CRSwNP had vitamin D deficiency

#### Symptom-based severity assessment

Using vitamin D status, patient-reported outcome measures have documented several associations. Bavi et al. (2019) reported moderate negative correlations between vitamin D levels and SNOT-22 scores, as determined by weighted scoring (r = -0.49, p < 0.001) (12). Similarly, Wang et al. (2019) studied CRSwNP and reported that vitamin D levels were negatively correlated with SNOT-22 scores (r = -0.378, p = 0.013) but positively correlated with 6-month postoperative improvement, as measured by the SNOT-22 weighted scoring (r = 0.567, p < 0.001). Importantly, 93% of patients diagnosed with CRSwNP were vitamin D-deficient (14) (**Table 4****).**

#### Associations with disease phenotypes

Investigation of various CRS phenotypes revealed a particularly pronounced deficiency in cases of polyposis. CRSwNP patients, as reported by Shanaki et al. (2017), had a mean vitamin D level of 12.11 ± 6.27 ng/mL, compared to 35.90 ± 17.19 ng/mL in the control group, resulting in a 36.27% reduction (p < 0.001) (9). Shrestha et al. (2022) reported vitamin D deficiency in 90% of primary and 100% of recurrent cases of polyposis, with mean vitamin D levels of 13.45 ± 9.12 ng/mL and 12.4 ± 8.47 ng/mL, respectively, p < 0.05 compared to 17.54 ± 7.94 ng/mL in controls (10) (**Table 4****).**

### Effects of taking vitamin D supplements

#### Improvement of symptoms

Intervention studies have demonstrated improvements, particularly with vitamin D supplementation. Kammili et al. (2024) noted a 34.4% improvement in SNOT-22 scores with daily supplementation of 400 IU, compared to 22.8% with placebo (p < 0.0001) (18). In addition, there was a 53.7% improvement in CT scores compared to 27.6% with the placebo (p < 0.0001). Stronger dosing regimens, as reported by Hashemian et al. (2020), using 4000 IU daily for one month, resulted in SNOT-22 scores of 16.25 ± 10.16 in the treatment group and 47.45 ± 13.55 in the placebo group at 6 months post-intervention (p < 0.001) (17) (**Table 4****).**

Ghazavi et al. (2023) reported that 50,000 IU weekly for 8 weeks significantly reduced SNOT-22 scores from 36.03 ± 10.71 to 29.90 ± 11.99 (p = 0.041) and reduced polyp recurrence to 24.1% compared to 51.6% in controls (OR = 0.298 [0.099-0.900], p = 0.032) (19). Baruah et al. (2020), using 60,000 IU weekly for 3 months, reported improving Total Nasal Symptom Scores from 11.94 to 0.33 with vitamin D and 11.96 to 2.29 with placebo (p < 0.0001) and increasing serum vitamin D from 12.31 ng/mL to 29.71 ng/mL (1) (**Table 4****).**

#### Surgical outcomes

Patients with severe disease underwent functional endoscopic sinus surgery (FESS), and their vitamin D levels were measured pre- and postoperatively. As reported by Kalińczak-Górna et al. (2021), postoperative levels were 27.67 ng/mL, representing an increase from the preoperative level of 23.01 ng/mL (p < 0.05) (13). This illustrates a reciprocal relationship in which both inflammation and vitamin D metabolism mutually influence each other (**Table 4****).**

#### Inflammatory markers

Sansoni et al. (2015), studying CRSwNP, found that some of the lowest levels of vitamin D were associated with some of the highest levels of inflammatory mediators, most notably RANTES and bFGF. RANTES (r = -0.612, p = 0.026) and bFGF (r = -0.578, p = 0.039) provided evidence suggesting possible explanations for the impact of vitamin D’s therapeutic effects (16) (**Table 4****).**

### Secondary outcomes

#### Quality of life assessment

Outcomes related to quality-of-life aspects, often assessed using the Sino-Nasal Outcome Test-22 (SNOT-22), consistently showed relationships with vitamin D status across various studies. Several observational studies have noted pronounced inverse relationships between serum vitamin D levels and SNOT-22 scores. Thakur and Potluri (2020) noted a strong negative correlation (r = -0.49, p < 0.001) (11), and Bavi et al. (2019) showed similar results in patients with chronic rhinosinusitis with nasal polyps (CRSwNP), with mean SNOT-22 scores of 89 ± 1.47 and a similar correlation (r = -0.49, p < 0.001) (12). Zand et al. (2020) noted a modest yet significant negative correlation (r = -0.22, p = 0.034) with mean SNOT-22 scores of 40.8 ± 17.6 (15) (**Table 5****).**

**Table 5:** Secondary outcome.

Author ID, country, year	Quality of life (SNOT-22)	Inflammatory markers	Postoperative outcomes	Recurrence rates	Adverse events
Thakur & Potluri, India, 2020 (11)	Significant inverse correlation with VD levels (r=-0.49, p<0.001)	Not measured	N/A	N/A	N/A
Kammili et al., India, 2024 (18)	Measured but specific values NA	IL-4, IL-5, IL-13 measured (values not specified in abstract)	N/A	Focused on disease severity	NA
Bavi et al., Iran, 2019 (12)	CRSwNP: 89 ± 1.47 Significant negative correlation with VD (r=-0.49, p<0.001)	Not measured	N/A	N/A	N/A
Shanaki et al., Iran, 2017 (9)	Measured (values in study)	IL-4: 2.9 ± 0.62 (CRSwNP) vs 6.35 ± 1.34 (controls), p<0.001 IL-5:208.63 ± 32.46 (CRSwNP) vs 146.63 ± 32.46 (controls), p<0.001 IL-13:20.49 ± 9.41 (CRSwNP) vs 23.63 ± 9.41 (controls)	N/A	N/A	N/A
Shrestha et al., India, 2022 (10)	Primary NP: 42.03 Recurrent NP: 46.07	IL-4: 2.96 ± 0.33 (Primary), 2.90 ± 0.62 (Recurrent) IL-5:238.24 ± 96.18 (Primary), 208.63 ± 32.46 (Recurrent) IL-13:16.83 ± 6.42 (Primary), 20.49 ± 9.41 (Recurrent)	N/A	Studied primary vs recurrent groups	NA
Hashemian et al., Iran, 2020 (17)	6 months post-op: VD3: 16.25 ± 10.16 Placebo: 47.45 ± 13.55 p<0.001	Not measured	Meltzer scores at 6m: VD3: 0.50 ± 0.60 Placebo: 2.65 ± 0.93 p<0.001	Significantly lower recurrence in VD3 group (p<0.001)	No adverse effects in VD3 group
Zand et al., Iran, 2020 (15) Iran	Mean SNOT-22: 40.8 ± 17.6 Negative correlation with vitamin D (r=-0.22, p=0.034)	NA		NA	NA
Kalińczak-Górna et al., Poland, 2021 (13)	Pre-op SNOT-22: Group 1: 30.33 Group 2: 31.80 Post-op SNOT-22: Group 1: 16.20 Group 2: 18.53	NA	Lund-Kennedy scale: Pre-op Group 1: 3.21, Group 2: 6.30 Post-op Group 1: 0.41, Group 2: 1.61	NA	NA
Sansoni et al., USA, 2015 (16)	Not measured	MCP-1: No significant correlation with vitamin D RANTES: Negative correlation in CRSwNP (r=-0.612, p=0.026) bFGF: Negative correlation in CRSwNP (r=-0.578, p=0.039)	NA	NA	NA
Ghazavi et al., Iran, 2023 (19)	Pre-op SNOT-22: Case: 54.38 ± 16.26 Control: 55.10 ± 15.62 Post-op SNOT-22: Case: 18.34 ± 8.30 Control: 25.19 ± 12.93 Change: 36.03 ± 10.71 vs 29.90 ± 11.99 (p=0.041)	NA	NA	NP recurrence: Case group: 24.1% Control group: 51.6%<br>(p=0.036)	NA
Baruah et al., India, 2020 (1)	Used TNSS instead of SNOT-22; Pre-treatment: 11.92; Post-treatment (3 months): 0.33 ± 0.61 (Vit D group) vs 2.29 ± 1.581 (placebo)	Not measured	N/A (medical treatment study, not surgical)	NA	NA
Wang et al., China, 2019 (14)	Pre-op SNOT-22 inversely correlated with Vit D levels (R=-0.378, p=0.013); Post-op improvement positively correlated with Vit D (R = 0.567, p<0.001)	Not measured	SNOT-22 improvement at 6 months correlated with baseline Vit D levels	NA	NA

Intervention studies and clinical trials have highlighted the therapeutic potential of vitamin D. Hashemian et al. (2020) documented marked changes in SNOT-22 scores six months after surgery (17). The vitamin D3 supplementation group’s scores were 16.25 ± 10.16, while the placebo group’s scores were 47.45 ± 13.55 (p < 0.001). Similarly, Ghazavi et al. (2023) reported greater benefits after surgery in the vitamin D group than in the control group, with SNOT-22 score changes of 36.03 ± 10.71 and 29.90 ± 11.99, respectively (p = 0.041) (19). Preoperative SNOT-22 scores also showed an inverse relationship with vitamin D levels, as reported by Wang et al. (2019) (R = -0.378, p = 0.013), and postoperative improvement was strongly positively associated with vitamin D status at baseline (R = 0.567, p < 0.001) (14) (**Table 5****).**

#### Inflammatory biomarkers

The association between vitamin D levels and inflammatory mediators has yielded different and often contradictory results across studies. For example, Shanaki et al. (2017) noted paradoxical results in CRSwNP patients with lower IL-4 levels (2.9 ± 0.62 vs. 6.35 ± 1.34, pg/mL, p < 0.001) and higher IL-5 levels (208.63 ± 32.46 vs. 146.63 ± 32.46 pg/mL, p < 0.001) while IL-13 levels were unchanged (9). In a more recent study, Shrestha et al. (2022) reassessed the inflammatory profiles of patients with polyps, noting similar IL-4 (2.96 ± 0.33 vs. 2.90 ± 0.62 pg/mL) and IL-5 (238.24 ± 96.18 vs. 208.63 ± 32.46 pg/mL) levels between the primary and recurrent polyp groups (10) (**Table 5****).**

Examining other inflammatory mediators, Sansoni et al. (2015) found a significant negative correlation with vitamin D in CRSwNP patients for both RANTES (r = -0.612, p = 0.026) and basic fibroblast growth factor (bFGF) (r = -0.578, p = 0.039), while monocyte chemoattractant protein-1 (MCP-1) had no significant correlation (**Table 5****).**

#### Postoperative clinical outcomes

Within the research on surgical results, Hashemian et al. (2020) demonstrated a significant improvement in Meltzer scores at 6 months for the vitamin D3 group (0.50 ± 0.60) compared to the placebo group (2.65 ± 0.93, p < 0.001) (13, 17). In the study conducted by Kalińczak-Górna et al. (2021), they noted changes in the Lund-Kennedy endoscopic scores, where Group 1 shifted from 3.21 to 0.41 and Group 2 from 6.30 to 1.61. Further supporting the link between baseline vitamin D levels and vitamin D metabolism (13), Wang et al. (2019) reported that improvements in SNOT-22 scores at 6 months were significantly associated with vitamin D levels, reinforcing the importance of preoperative vitamin D levels (14) (**Table 5****).** Although postoperative increases in vitamin D3 levels have been reported, the biological mechanisms underlying this observation remain unclear and require further mechanistic investigation.

#### Recurrence rates

Two studies provided compelling evidence for the role of vitamin D in preventing disease recurrence. Ghazavi et al. (2023) reported recurrence rates of 24.1% in the vitamin D supplementation group versus 51.6% in the control group (p = 0.036), representing a greater than 50% reduction in recurrence risk (19). Hashemian et al. (2020) similarly documented significantly lower recurrence rates in the vitamin D3 group compared to the placebo group (p < 0.001), although specific percentages were not provided (17). Shrestha et al. (2022) were more concerned with the comparative analysis of the primary versus recurrent groups than with prospective recurrence rates (10) (**Table 5****).**

#### Safety profile

The safety profile of vitamin D supplementation appeared to be favorable across all intervention studies. Hashemian et al. (2020) explicitly reported no adverse effects in the vitamin D3 supplementation group (17). Most studies either reported no adverse events or did not include safety data in their outcomes, suggesting that the intervention was generally well tolerated. No serious adverse events were associated with vitamin D supplementation in any of the included studies (**Table 5****).**

#### Non-surgical management treatment response

Baruah et al. (2020) analyzed a case of medical management utilizing the TNSS score rather in lieu of SNOT-22 and reported remarkable improvements of baseline score 11.92 to 0.33 ± 0.61 in the treatment (vitamin D group) vs 2.29 ± 1.581 in the placebo group at 3 months post-treatment, demonstrating a non-surgical management approach with vitamin D (1) (**Table 5****).**

### Meta-analysis of reported outcomes

**Figure 2:**
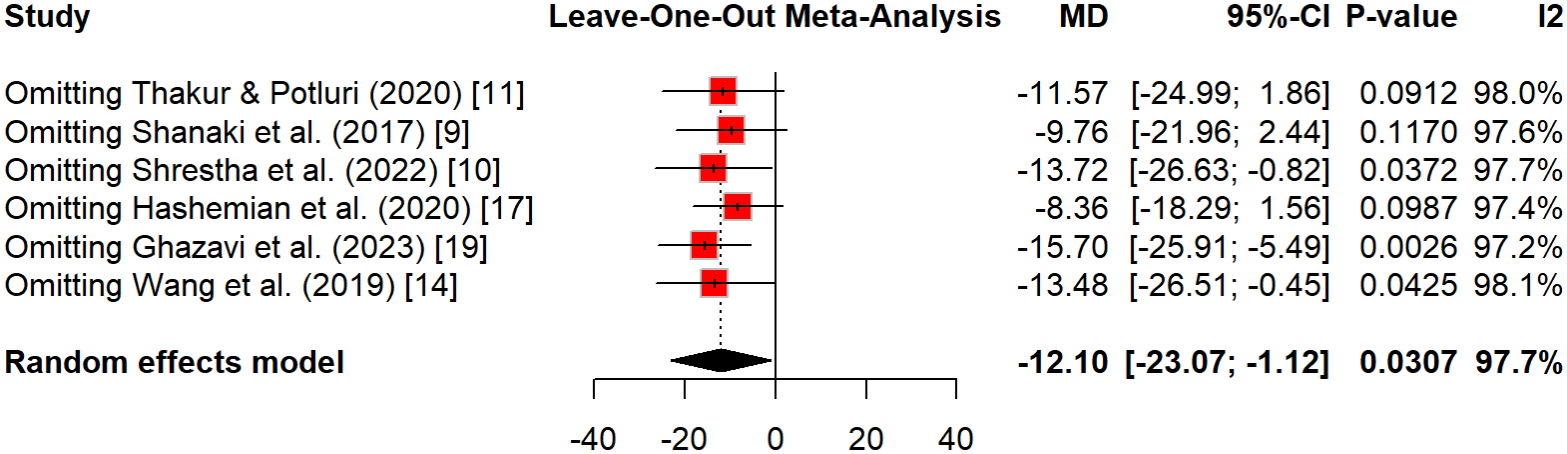
Sensitivity analysis for comparison of vitamin D level (ng/mL).

Our meta-analysis demonstrated a significant reduction in serum vitamin D levels among patients who had chronic rhinosinusitis (CRS) of any type as compared to the controls or placebo group. Notably, the pooled mean difference for this effect size was −12.09 ng/mL with 95% CI of −23.07 to −1.12. This indicated that CRS patients had substantially lower levels of vitamin D. This effect was statistically significant (p = 0.0307) ***(*****Figure 1*****)***. However, the heterogeneity was very high (I² = 97.7%), which suggested that there is considerable variability across studies. In order to find source of heterogeneity, we performed sensitivity meta-analysis, and **Figure 2** shows that omission of none of the included studies didn’t reduce heterogeneity.

**Figure 3:**
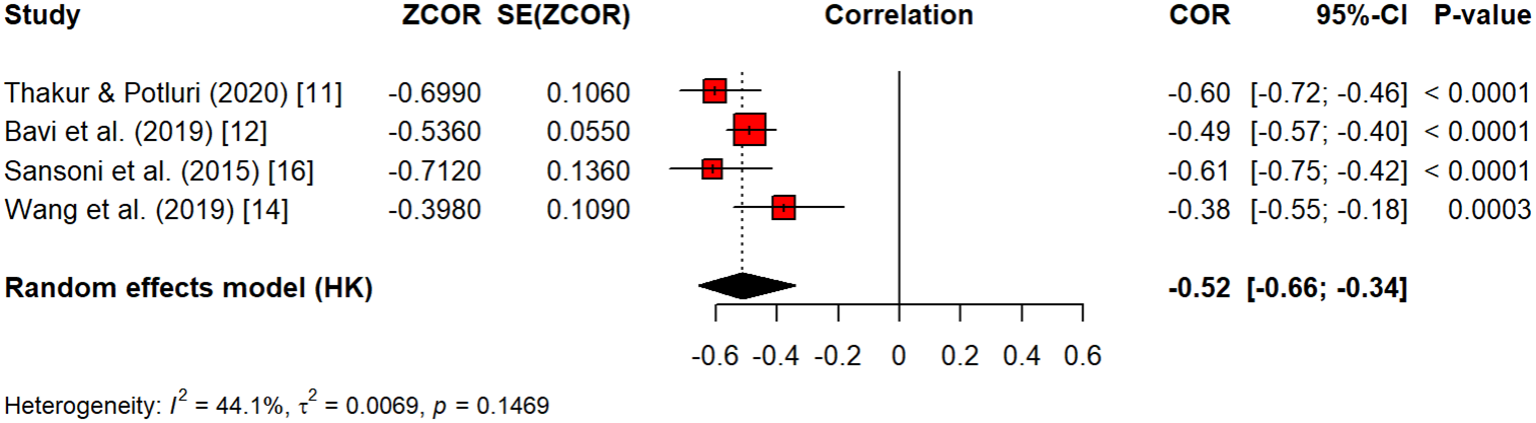
Correlation of vitamin D levels with the severity of diseases.

**Figure 4:**
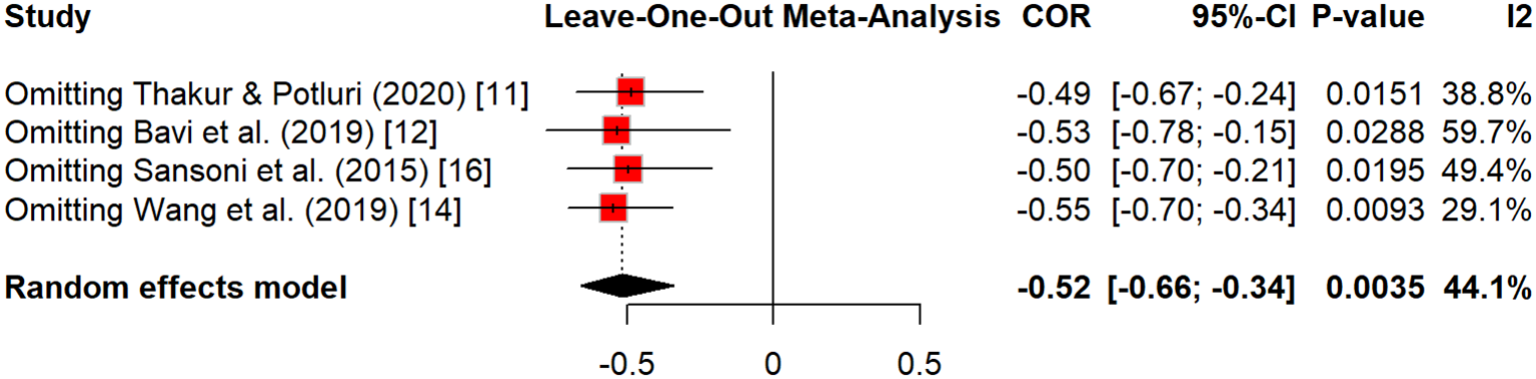
Sensitivity analysis for the correlation of vitamin D levels with the severity of diseases.

Furthermore, our meta-analysis also evaluated the correlation between vitamin D levels and disease severity, which revealed a significant inverse association. The pooled correlation coefficient under the random-effects model was −0.52 with 95% CI of −0.66 to −0.34. This indicated that lower levels of vitamin D were significantly associated with increased severity of CRS. Notably, this effect was statistically significant (p = 0.0035). The heterogeneity for this effect size was moderate with I² of 44.1% which supported the reasonable consistency of findings across studies and strengthened the reliability of this finding ***(*****Figure 3*****)***. Furthermore, omission of Wang (2019) (14) significantly reduced heterogeneity with I² of 29.1% as shown in **Figure 4**.

**Figure 5:**
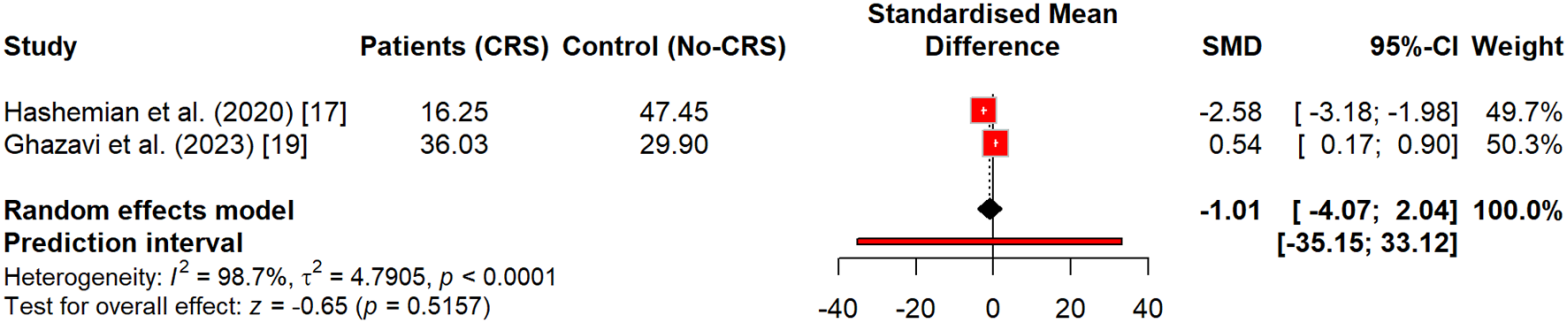
Comparison of QoL (SNOT-22) between CRS patients and comparison group.

Moreover, the meta-analysis of quality-of-life outcomes with the SNOT-22 score didn’t show any statistically significant difference between the vitamin D and control groups ***(*****Figure 5*****)***. The pooled standardized mean difference (SMD) was −1.01 with 95% CI of −4.07 to 2.04 (p = 0.5157). Notably, heterogeneity was very high (I² = 98.7%), and only two studies were included which limited the robustness of this result.

**Figure 6:**
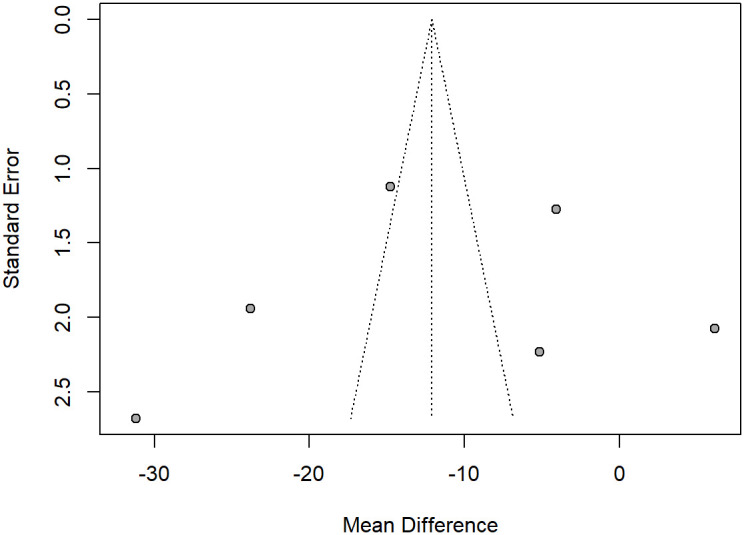
Assessment of risk of bias with funnel plot.

**Figure 6** shows the funnel plot for the assessment of potential publication bias in the included studies. Notably, the distribution of the included studies appears asymmetric, and more data points were scattered on one side of the pooled effect estimate. Ideally, all the studies should be symmetrically distributed around the central line. This imbalance suggested that there is possible publication bias or small-study effects. Additionally, the widespread of the points shows substantial heterogeneity among studies. The limited number of studies further reduces the reliability of the visual interpretation.

## Discussion

Our systematic review demonstrated that vitamin D levels in patients with CRS were significantly lower than those in controls (12–15 ng/mL versus 29–36 ng/mL), reinforcing the existing systematic evidence. We also found support from Li et al.’s (2019) meta-analysis, which reported a weighted mean difference of -7.80 ng/mL in vitamin D levels in patients with CRS compared to controls (20). In conjunction with several systematic reviews that consistently document a prevalence of vitamin D deficiency exceeding 90% in patients with nasal polyposis, the evidence supports our findings (7, 21). The strong correlations between vitamin D deficiency and disease severity markers (CT scores: r = -0.604 to -0.66; endoscopic scores: r = -0.595 to -0.71) strengthen the argument that vitamin D serves as a reliable biomarker in CRS and substantiate the notion that vitamin D may be a therapeutic target.

Our meta-analysis further strengthens these findings by quantitatively demonstrating significantly lower vitamin D levels in patients with CRS compared to controls, along with a moderate inverse correlation between vitamin D levels and disease severity. Despite the presence of substantial heterogeneity in pooled vitamin D levels, the consistency in the direction of effect across studies supports a true biological association. The absence of a statistically significant pooled effect for SNOT-22 outcomes may be attributed to variability in study designs, limited sample sizes, and differences in intervention protocols rather than a lack of clinical benefit.

The effectiveness demonstrated in our study, where SNOT-22 scores improved by 34.4% and 22.8% in the placebo group, and CT scores improved by 53.7% compared to 27.6%, exceeds what is typically observed in case studies of other chronic inflammatory diseases. From asthma studies, we observed a 36% improvement in exacerbations (22), and with inflammatory bowel disease, a 30% reduction in the rate of surgeries (23). However, the extent of improvements in CRS is remarkably stratified relative to other diseases, suggesting a particularly responsive pathophysiological framework for CRS. This differential responsiveness may be attributed to the localized, high-density expression of vitamin D receptors in the sinonasal epithelium, as well as the significant role of vitamin D metabolism at the local level in regulating mucosal balance and inflammation.

Understanding the immunomodulatory functions of vitamin D provides a clearer explanation of our clinical insights. In this regard, sinonasal tissues exhibit dysregulated local vitamin D metabolism, characterized by decreased active 1,25(OH)_2_D_3_ production due to reduced CYP27B1 expression and increased catabolic CYP24A1, which accelerates vitamin D breakdown (21, 24). This creates a state of ‘local vitamin D deficiency’ that can persist even when systemic levels are sufficient. This explains the unwillingness of some patients to respond to standard supplementation and indicates the need to explore more directly modified local delivery methods. Recent evidence further supports the concept that dysregulated local vitamin D metabolism, including reduced CYP27B1 expression, altered CYP24A1 activity, and impaired vitamin D receptor (VDR) signaling, may contribute to the pathophysiology of CRSwNP by limiting local availability and biological activity of active vitamin D (25).

Calcitriol’s suppression of pro-inflammatory chemokines RANTES and bFGF, which our review noted was inversely associated with vitamin D levels, provides direct mechanistic links to diminished eosinophil recruitment and tissue remodeling (16, 25). Through the multifaceted epithelial barrier integrity effects of vitamin D, which include upregulation of tight junction proteins (occludin, claudin-14, ZO-1), antimicrobial peptides (cathelicidin, β-defensins), and modulation of NF-κB and MAPK signaling pathways, the vitamin D effects on the disease are multifactorial and explain the observed improvement (26, 27). Vitamin D-deficient patients with CRSwNP exhibit a correlation with the phenomenon of vitamin D deficiency, which is associated with promoting Th2-skewed eosinophilic polypoid disease inflammation. Emerging evidence also suggests that vitamin D may enhance responsiveness to corticosteroid therapy through modulation of inflammatory signaling pathways and immune regulation, potentially providing complementary therapeutic benefits in patients with CRSwNP, although further clinical studies are needed to establish the significance of this interaction (25).

Recent research has further uncovered mechanisms, including vitamin D’s role in trained immunity via epigenetic reprogramming and its involvement in controlling pro-resolving mediators that terminate inflammation, as well as its impact on the sinonasal microbiome (28). These findings suggest that vitamin D is not only an anti-inflammatory agent but also a primary regulator of immune response balance in mucous membranes. Vitamin D deficiency creates several host factors that interact with and sustain chronic sinonasal inflammation (29).

Despite systematic reviews and mechanistic study findings supporting the use of vitamin D, our research revealed a striking absence of vitamin D recommendations in major clinical guidelines. There are no provisions for the assessment or supplementation of vitamin D in the management of CRS in the American Academy of Otolaryngology-Head and Neck Surgery guidelines, the Fokkens et al. (2020) European Position Paper on Rhinosinusitis and Nasal Polyps 2020, or the International Consensus Statement on Allergy and Rhinology (30). This discrepancy between the available evidence and practice highlights a significant gap in the utilization of safe and cost-effective interventions to improve patient care.

The absence of defined protocols for vitamin D assessment, testing, target thresholds and levels, dosing, and its incorporation into existing therapies results in significant gaps in care, potentially undermining patient outcomes. There is a lack of endorsement from a professional society for a 60,000 IU weekly regimen for 12 weeks, aimed at surpassing a level of 30 ng/mL, which limits its adoption. This concern is amplified by the overwhelming dominance of economic models for vitamin D supplementation, which grant better outcomes and reduced costs through lower healthcare utilization, with pragmatic trials demonstrating reduced hospital and emergency visitation rates (31, 32).

Within the scope of other chronic inflammatory disorders, CRS stands out as perhaps the most responsive to vitamin D intervention among inflammatory disorders responsive to vitamin D. The 53.7% improvement in CT scores and the 51.6% to 24.1% reduction in recurrence rates are far more responsive than those seen in asthma, inflammatory bowel disease, and rheumatoid arthritis (33, 34). This remarkable responsiveness is likely due to the intersection of multiple vitamin D-dependent pathways in sinonasal diseases, including epithelial barrier malfunction, mucosal immunity imbalance, suboptimal antimicrobial defenses, and altered tissue remodeling processes (34, 35).

CRS treatment with vitamin D has a markedly favorable safety profile compared to conventional CRS treatments. Systemic corticosteroids carry significant adverse effects, biologics require careful patient selection and monitoring, and surgical interventions carry inherent risks due to the nature of the procedures. The safety profile demonstrated in our review and the broader literature, with no serious adverse events at doses up to 10,000 IU daily and rare toxicity with standard weekly replacement regimens of50,000 IU, makes vitamin D a promising add-on approach to lessen reliance on other, more aggressive treatments (36).

## Limitations

When interpreting our results, several limitations should be considered. The heterogeneity of vitamin D supplementation, with some participants receiving daily oral doses and others receiving monthly intramuscular injections, makes direct comparisons and optimizations challenging. Some studies’ baseline vitamin D levels were the participants’ vitamin D-replete; hence, those participants would be less likely to demonstrate treatment benefit, which adds to the discrepancy (8, 37, 38). The predominance of observational studies (9 out of 12) hinders causal inference, although the consistency of associations across different populations is reassuring. However, geographic latitude, sun exposure, dietary intake, and seasonal variation are important covariates that have been reported inconsistently across studies. Some response heterogeneity can be explained by genetic polymorphisms that impact vitamin D metabolism, particularly those in the vitamin D receptor (VDR) genes BsmI and TaqI, as well as CYP2R1 and DBP; however, such genetic factors have been rarely studied (39–41). The insufficient follow-up duration in most studies likely underappreciates the long-term benefits, especially in preventing recurrence. Moreover, most studies analyzed the concentration of vitamin D in the blood without evaluating the metabolism of the sinonasal tissues, which may have contributed to the omission of some important pathophysiological mechanisms (25). The meta-analysis was limited by substantial heterogeneity across studies, differences in supplementation regimens, and a small number of studies for certain outcomes (e.g., SNOT-22), which may affect the robustness of pooled estimates. Additionally, publication bias cannot be excluded, as unpublished studies with negative findings may not be represented in the available literature.

## Future research recommendations

The integration of precision medicine technologies with the role of vitamin D in CRS presents novel and transformative research opportunities. Some priority areas include the design of supplementation protocols that integrate genetic phenotyping of vitamin D metabolism with biomarker-adequate supplementation, the design of optimal therapeutic windows using tissue-specific metabolomic profiling, and the exploration of synergistic combinations of biologics and microbiome-targeted therapies. The complex effects of vitamin D in the sinonasal microenvironment can be elucidated using patient-derived organoids and tissue-specific spatial metabolomics. This approach can also predict a patient’s response to personalized treatment before it is administered. Future studies should also investigate phenotype-specific effects of vitamin D, particularly in patients with CRSwNP, given the distinct inflammatory mechanisms and potential differences in therapeutic responsiveness between CRS phenotypes.

Recent randomized clinical evidence demonstrated that calcifediol added to saline nasal irrigation with budesonide significantly improved SNOT-22 scores, Lund-Kennedy endoscopic scores, and serum 25(OH)D levels compared with placebo, further supporting the need for larger, well-designed trials to define the role of vitamin D-based interventions in CRS management and identify patients most likely to benefit from treatment (42).

Topical Vitamin D for treating local metabolic dysregulation, anti-inflammatory vitamin D analogs, and omega-3 fatty acids to stimulate pro-resolving mediators warrant further research. Flexible clinical protocols permitting modifications in response to biomarker feedback, N-of-1 personalized optimization, and diversity-focused pragmatic effectiveness frameworks will expedite the shift to clinical application. Translational research addressing vitamin D deficiency, which is particularly prevalent in patients with CRS, highlights the socially inequitable frameworks that these patients face. CRS and vitamin D are intricately linked to socioeconomic and ethnic variations in vitamin D receptor polymorphisms and geographic sun exposure, necessitating targeted public health interventions.

## Conclusion

This systematic review and meta-analysis demonstrate that vitamin D deficiency is strongly associated with the development and increased severity of chronic rhinosinusitis (CRS), particularly among patients with nasal polyps. Consistent inverse correlations were observed between serum vitamin D levels and key disease severity markers, including radiological, endoscopic, and patient-reported outcomes. Quantitative meta-analysis further supports these findings, confirming significantly lower vitamin D levels in CRS patients and a moderate inverse relationship with disease severity.

Evidence from interventional studies indicates that vitamin D supplementation is associated with meaningful improvements in symptom severity, quality of life, and radiological outcomes, along with a substantial reduction in recurrence rates. Importantly, supplementation demonstrated a favorable safety profile, with no significant adverse events reported. These therapeutic effects are likely mediated through multiple mechanisms, including modulation of inflammatory pathways, enhancement of epithelial barrier function, and regulation of local immune responses.

The pronounced responsiveness of CRS to vitamin D suggests a disease-specific pathophysiological susceptibility, positioning vitamin D as a promising adjunctive therapeutic strategy. Despite accumulating evidence, routine assessment and correction of vitamin D deficiency remain underutilized in current clinical practice, representing a critical gap between evidence and implementation.

Future research should focus on identifying patient subgroups most likely to benefit from supplementation and establishing standardized, evidence-based dosing protocols. High-quality randomized controlled trials are needed to inform clinical guidelines and optimize the integration of vitamin D into comprehensive CRS management strategies.

## Data Availability

The original contributions presented in the study are included in the article/supplementary material, further inquiries can be directed to the corresponding author/s.
